# Complete Genome Sequence of *Actinomyces hongkongensis* HKU8^T^ Isolated from Human Blood

**DOI:** 10.1128/genomeA.01650-16

**Published:** 2017-04-06

**Authors:** Yan-xia Gao, Yu-yan Zhou, Ying Xie, Man Wang, Shu-juan Wang, Shi-gang Shen

**Affiliations:** a Hebei Provincial Institute for Drug Control, Shijiazhuang, China; b Changchun University of Technology, Changchun, China; c Hebei University, Baoding, China

## Abstract

Members of the genus *Actinomyces* are strongly associated with human diseases. We present here the complete genome sequence of *Actinomyces hongkongensis* HKU8^T^, which consists of one circular chromosome. The strain characteristically contains various genes encoding for enzymes involved in arylamidase utilization.

## GENOME ANNOUNCEMENT

There has been increasing recognition of the importance of coryneform bacteria as opportunistic human pathogens (1). As a result of increased medical interest in such organisms, combined with intensified taxonomic investigations, a number of new bacteria have been described in recent years (2). Members of the genus *Actinomyces* are known to be indigenous bacteria, colonizing mucosal surfaces of humans and other homeothermic animals. They are strongly associated with human diseases, such as urosepsis, oral cervicofacial actinomycosis, thoracic actinomycosis, abdominal actinomycosis, pelvic actinomycosis, central nervous system actinomycosis, musculoskeletal actinomycosis, and disseminated actinomycosis. Currently, the *Actinomyces* genus consists of over 30 species, with a number of novel *Actinomyces* species described in recent years (3–6).

*Actinomyces hongkongensis* HKU8^T^ (= DSM 15629T = LMG 21939^T^) was isolated from human blood (7). We determined the complete genome sequence of *A. hongkongensis* HKU8 using the Illumina HiSeq 2000 Platform (Illumina, San Diego, CA, USA). We constructed one small-insert (500 bp) and three large-insert (6 kb, 8 kb, 12 kb) genomic DNA libraries and generated a total of 120,126,078 paired-end sequence reads (about 6,000-fold coverage) for* A. hongkongensis* HKU8^T^. Data were initially assembled with the SOAPdenovo program, and the genome was finished using gap-closing software in the SOAP packages. An initial set of predicted protein-coding genes was identified using Glimmer version 3.0. Genes consisting of <120 bp (bp) and those containing overlaps were eliminated. The tRNA genes were predicted by tRNAscan-SE, and the rRNA genes were detected by a BLASTn search using known *Bifidobacterium* rRNA sequences as queries. The genome sequence of *A. hongkongensis* HKU8^T^ consists of one circular chromosome of 2,141,493 bp with no plasmid.

The chromosome contains 1,740 predicted protein-coding genes and 46 RNA genes—1,532 (88%), 1,498 (86%), and 1,406 (81%) of which were conserved in the genomes of *Actinomyces* sp. strain F0588 (NZ_CP012590), *A. radicidentis* strain CCUG 36733 (NZ_CP014228), and *A. meyeri* strain W712 (NZ_CP012072), respectively. The remaining genes were dominated by hypothetical proteins or proteins of unknown function. The alignment of both genomes showed largely colinearity. Genes encoding for the oxidation/fermentation of arabinose, glucose, mannose, raffinose, trehalose, xylose, α/β-galactosidase, and α/β-glucosidase were not detected. The remaining genes contained several arylamidase-utilization gene clusters, which consist of arginine dehydrogenase, alkaline phosphatase, alanine arylamidase, leucine arylamidase, phenylalanine arylamidase, and proline arylamidase. This complete genome sequence will be useful for comparative genome analyses of *A. hongkongensis* strains.

### Accession number(s).

This whole-genome shotgun project has been deposited at GenBank under the accession number CP017298. The version described in this paper is the first version, CP017298.1.

## References

[1] Lawson PA, Falsen E, Akervall E, Vandamme P, Collins MD 1997 Characterization of some *Actinomyces*-like isolates from human clinical specimens: reclassification of *Actinomyces suis* (Soltys and Spratling) as *Actinobaculum suis* comb. nov. and description of *Actinobaculum schaalii* sp. nov. Int J Syst Bacteriol 47:899–903. doi:10.1099/00207713-47-3-899. 9226926

[2] Flynn AN, Lyndon CA, Church DL 2013 Identification by 16s rRNA gene sequencing of an *Actinomyces hongkongensis* isolate recovered from a patient with pelvic actinomycosis. J Clin Microbiol 51:2721–2723. doi:10.1128/JCM.00509-13. 23698532 PMC3719606

[3] Hoyles L, Pascual C, Falsen E, Foster G, Grainger JM, Collins MD 2001 *Actinomyces marimammalium* sp. nov., from marine mammals. Int J Syst Evol Microbiol 51:151–156. doi:10.1099/00207713-51-1-151. 11211252

[4] Hoyles L, Falsen E, Pascual C, Sjödén B, Foster G, Henderson D, Collins MD 2001 *Actinomyces catuli* sp. nov., from dogs. Int J Syst Evol Microbiol 51:679–682. doi:10.1099/00207713-51-2-679. 11321114

[5] Hoyles L, Falsen E, Holmström G, Persson A, Sjödén B, Collins MD 2001 *Actinomyces suimastitidis* sp. nov., isolated from pig mastitis. Int J Syst Evol Microbiol 51:1323–1326. doi:10.1099/00207713-51-4-1323. 11491328

[6] Hoyles L, Falsen E, Foster G, Collins MD 2002 *Actinomyces coleocanis* sp. nov., from the vagina of a dog. Int J Syst Evol Microbiol 52:1201–1203. doi:10.1099/00207713-52-4-1201. 12148628

[7] Woo PCY, Fung AMY, Lau SKP, Teng JLL, Wong BHL, Wong MKM, Hon E, Tang GWK, Yuen K 2003 *Actinomyces hongkongensis*, sp. nov.—a novel actinomyces species isolated from a patient with pelvic actinomycosis. Syst Appl Microbiol 26:518–522. doi:10.1078/072320203770865819. 14666979

